# Comprehensive Evaluation of 1H-Isoindole-1,3(2H)-Dione Derivatives: Pharmacokinetic Studies and Analgesic Potential in Various Pain Models

**DOI:** 10.3390/ijms26136026

**Published:** 2025-06-23

**Authors:** Anna Dziubina, Dominika Szkatuła, Łukasz Szczukowski, Małgorzata Szafarz, Anna Rapacz

**Affiliations:** 1 Department of Pharmacodynamics, Faculty of Pharmacy, Jagiellonian University Medical College, 9 Medyczna St., 30-688 Krakow, Poland; anna.dziubina@uj.edu.pl; 2 Department of Medicinal Chemistry, Wrocław Medical University, 211 Borowska St., 50-556 Wrocław, Poland; dominika.szkatula@umw.edu.pl (D.S.); lukasz.szczukowski@umw.edu.pl (Ł.S.); 3 Department of Pharmacokinetics and Physical Pharmacy, Faculty of Pharmacy, Jagiellonian University Medical College, 9 Medyczna St., 30-688 Krakow, Poland; malgorzata.szafarz@uj.edu.pl

**Keywords:** neuropathic pain, oxaliplatin, streptozotocin, carrageenan-induced edema, analgesic activity, 1H-isoindole-1,3(2H)-dione

## Abstract

The study investigated the antinociceptive effects of four compounds (F1–F4) based on a 1H-isoindole-1,3(2H)-dione core, using various in vivo pain models—tonic (formalin test), neurogenic (capsaicin and glutamate tests), neuropathic (oxaliplatin-induced model of peripheral neuropathy as well as the streptozotocin-induced model of painful diabetic neuropathy), and inflammatory (carrageenan-induced). Pharmacokinetic parameters were also assessed. In the capsaicin test, F1, F2, and F4 (5–20 mg/kg) significantly reduced pain, while compound F3 was only active at 20 mg/kg. In the glutamate test, F1, F2, and F3 (5–20 mg/kg) demonstrated the most pronounced effect. In phase I of the formalin test, compounds F1 and F2 were active at doses of 5 and 10 mg/kg, respectively, while F3 and F4 exhibited activity only at the 20 mg/kg dose. In phase II, a dose-dependent reduction in pain was observed, with the weakest effect noted at F4. At a dose of 20 mg/kg, the compounds significantly reduced edema and carrageenan-induced pain, but to a lesser extent than ketoprofen. The compounds tested (10 mg/kg) showed significant anti-allodynic activity in the oxaliplatin- and streptozotocin-induced neuropathy pain models. All compounds demonstrated favorable pharmacokinetic results. The results of this study indicate that the compounds have a broad analgesic spectrum of activity.

## 1. Introduction

Phthalimide is a well-established pharmacophoric scaffold containing an 1Hisoindoline-1,3 (2H)-dione core with an imide group. Notably, unlike the glutaramide moiety present in thalidomide, the phthalimide structure is not associated with teratogenicity or other severe adverse effects, rendering it a safer alternative in the development of bioactive molecules. Numerous studies have highlighted the pharmacological potential of phthalimide derivatives, which demonstrate a broad spectrum of activities, including anti-inflammatory, analgesic, antitumor, antimicrobial, and anticonvulsant effects, among others [1]. This favorable safety profile has facilitated the extensive use of the phthalimide moiety in the rational design and synthesis of novel therapeutic agents. Thalidomide, the most well-known compound within this chemical class, functions as a potent inhibitor of tumor necrosis factor-alpha (TNF-α) and interleukin-6 (IL-6). Despite its historical notoriety, thalidomide is currently approved for the treatment of *erythema nodosum leprosum* and multiple myeloma [2]. More recent and considerably safer analogues, such as lenalidomide and pomalidomide, belong to the class of immunomodulatory imide drugs and are widely used in oncology, particularly in the treatment of multiple myeloma and other hematological malignancies [3,4]. Another notable phthalimide-based compound, apremilast, has been identified as a selective phosphodiesterase-4 (PDE4) inhibitor. By modulating the production of pro- and anti-inflammatory cytokines, apremilast has demonstrated therapeutic efficacy in the management of psoriatic arthritis and has been approved for clinical use [5]. Taken together, these findings underscore the significance of the phthalimide scaffold in drug discovery and development, offering a structurally attractive and pharmacologically versatile platform for the design of novel therapeutics.

**Figure 1:**
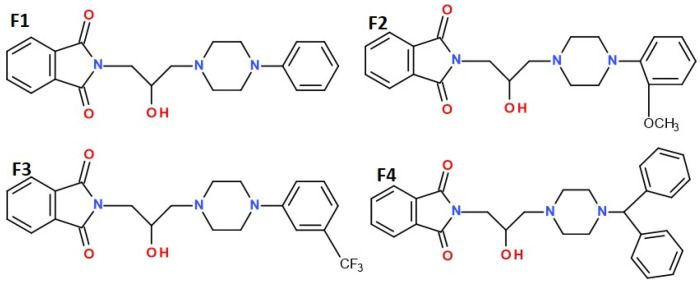
Structures of the studied compounds: F1, F2, F3, and F4.

The present study continues our previous research on N-aryl piperazine alkyl phthalimide derivatives [6]. Four compounds, F1–F4, differing in the pharmacophore structure within the phenyl ring of the 2-hydroxy-3-(4-aryl-1-piperazinyl)propyl moiety attached to the imide nitrogen (R; F1–F3) and the 4-benzhydryl analog (F4) [Figure 1], were shown to be non-cytotoxic in vitro in RAW 264.7 cells and significantly reduced COX-2 expression in LPS-stimulated macrophages, indicating at least partial anti-inflammatory potential. All tested compounds formed stable complexes with major plasma proteins—albumin, orosomucoid, and gamma globulin [7]. In initial in vivo screening (writhing and hot plate tests), compounds F1–F4 significantly inhibited peripheral nociception, while compounds F1–F3 also exhibited weaker activity in a central (supraspinal) pain model [6]. Compound F4 was inactive in the hot plate test and did not impair locomotor activity within the tested dose range. Compounds F1 and F3 showed mild sedative effects only at the highest analgesic doses, whereas F2 exhibited dose-dependent suppression of spontaneous locomotor activity. None of the compounds induced motor deficits.

The aim of this study was to further evaluate the analgesic activity of selected phthalimide derivatives in a broader range of experimental pain models. Specifically, we assessed the antinociceptive effects of F1–F4 in models representing tonic, neurogenic, acute inflammatory, and chemotherapy- or diabetes-induced neuropathic pain. These models involve different types of algogenic stimuli (mechanical, thermal, chemical) that produce similar pain symptoms through distinct mechanisms of action.

Chemically induced acute, neurogenic pain, was modeled using the capsaicin test and the early phase of the formalin test. The late phase of the formalin test, as well as carrageenan-induced inflammation, were used to analyze persistent pain and hyperalgesia associated with peripheral tissue injury and aseptic inflammation. Neuropathic pain associated with diabetes or dysfunction of the peripheral or central nervous system was induced by single administrations of streptozotocin and oxaliplatin, respectively. A preliminary pharmacokinetic study was also performed for compounds F1–F4 to facilitate interpretation of their in vivo pharmacological effects.

## 2. Results

### 2.1. Pharmacokinetic Results

Pharmacokinetic (PK) parameters calculated using non-compartmental analysis based on the serum or brain concentration–time data after single *i.p.* administration of the investigated compounds are summarized in Table 1.

All studied compounds reached maximal concentration (C_max_) at the first sampling point (5 min after administration); however, the values of C_max_ were quite different. The highest concentrations were achieved by compounds F1 and F2 (595.75 µg/L and 699.75 µg/L, respectively) while compounds F3 and F4 reached concentrations equal to 210.23 and 130.58 µg/L, respectively. Compound F4 had the highest clearance and the lowest area under the concentration–time curve extrapolated to infinity (AUC0-inf). The high clearance was due to the high volume of distribution (V_d_) since the elimination constant for all studied compounds was similar. The high V_d_, in turn, is derivative of low plasma concentration and there might be two explanations for this situation. Either compound F4 has a much better ability to cross biological barriers, compared to the others, or it has the lowest bioavailability after *i.p.* administration. Since the brain/serum partitioning coefficient (K_brain/serum_) for compound F4 is similar to those calculated for compounds F1 and F3, the most likely explanation is low bioavailability. All of the studied compounds cross the blood/brain barrier (BBB). The highest brain concentration was achieved by compound F1, and the lowest by compound F4. The half-life for all studied compounds was similar and ranged from 0.75 h for compound F2 to 1.26 h for compound F3.

### 2.2. In Vivo Pharmacological Study Results

#### 2.2.1. Antinociceptive Activity in the Formalin Test

In the formalin test, the compounds F1, F2, F3, and F4 at a dose range of 5 to 20 mg/kg after *i.p.* administration were tested.

**Figure 2:**
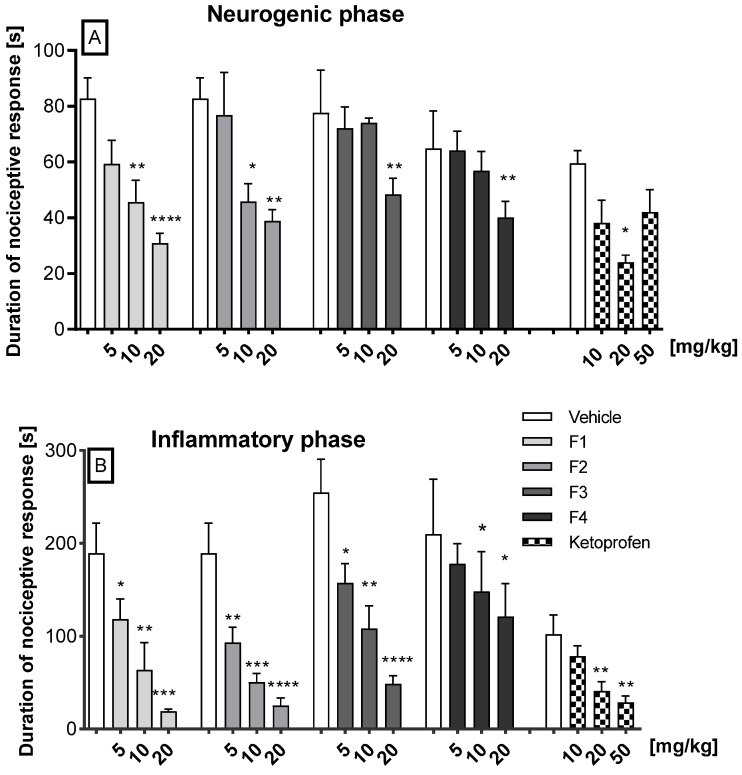
The impact of F1, F2, F3, F4, and ketoprofen on the duration of licking and biting behavior was assessed in the first (neurogenic) phase (0–5 min after formalin injection) (**A**) and in the second (inflammatory) phase (15–30 min after formalin injection) (**B**) of the formalin test in mice. The test compound or vehicle were administered 30 min *i.p.* before the test. The results are presented as bar plots showing the mean ± SEM. Statistical analysis: one-way analysis of variance (ANOVA), followed by Ṧidak’s multiple comparison post hoc test. Significance vs. vehicle * *p* < 0.05; ** *p* < 0.01; *** *p* < 0.001; **** *p* < 0.0001, n = 8 mice per group.

In the neurogenic phase, intraperitoneal administration of F1 and F2 at the doses of 10 and 20 mg/kg significantly diminished the pain reaction, for F1 by 44.9% (*p* = 0.0023) and 62.7% (*p* < 0.0001), and for F2 by 44.6% (*p* = 0.0196) and 53% (*p* = 0.0047), respectively. In the same phase, compounds F3 and F4 at the dose of 20 mg/kg displayed antinociceptive effects, as they significantly decreased the duration of the licking response by 41.6% (*p* = 0.0021) and 48.3% (*p* = 0.0020), respectively. Compounds F1, F2, F3, and F4 at the dose of 5 mg/kg, and also F3 and F4 at the dose of 10 mg/kg did not attenuate the nociceptive response in this phase in a statistically significant manner. Ketoprofen, used as reference, only at 20 mg/kg, statistically significantly reduced pain behavior by 59.7% compared to control (*p* = 0.0016). The other doses did not show a statistically significant reduction in neurogenic pain (Figure 2A).

In the second (inflammatory) phase of the formalin test, dose-dependent and significant antinociceptive activity was observed for compounds F1, F2, and F3 when compared to the vehicle. Thus, F1 reduced the pain response at doses of 5, 10, and 20 mg/kg by 37% (*p* = 0.045), 66% (*p* = 0.0078), and 90% (*p* = 0.0003), respectively; F2 diminished the pain reaction at doses of 5, 10, and 20 mg/kg by 51% (*p* = 0.0319), 73% (*p* = 0.0009), and 87% (*p* < 0.0001), respectively; and F3 by 34.5% (*p* = 0.040), 57.5% (*p* = 0.0017), and 80.9% (*p* < 0.0001), respectively. Compound F4, at the doses of 10 mg/kg and 20 mg/kg, diminished pain by 29.5% and by 42% (*p* < 0.05), respectively. Ketoprofen showed a dose-dependent analgesic effect. At a dose of 10 mg/kg, a 23.2% reduction in pain was observed, which did not reach statistical significance. In contrast, doses of 20 mg/kg and 50 mg/kg resulted in a reduction in pain of 59.9% (*p* = 0.0041) and 71.8% (*p* = 0.0014), respectively. The precise data are illustrated in Figure 2B

#### 2.2.2. Antinociceptive Activity in the Glutamate- and Capsaicin-Induced Pain Tests

**Figure 3:**
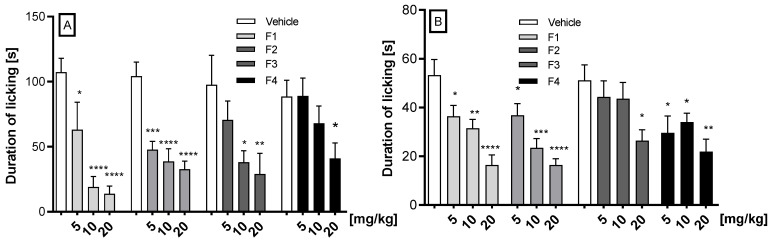
The effect of F1, F2, F3, and F4 compounds administered *i.p.* 30 min before the test on the duration of licking and biting behavior in the glutamate (**A**) and the capsaicin (**B**) tests. Data are expressed as mean ± SEM. Statistical analysis: one-way (ANOVA), followed by Dunnett’s post hoc test; Significance vs. vehicle * *p* < 0.05; ** *p* < 0.01, *** *p* < 0.001; **** *p* < 0.0001, n = 8 mice per group.

In the glutamate-induced pain test, the compounds F1, F2, F3, and F4 at a dose range of 5 to 20 mg/kg after *i.p* administration were tested (Figure 3A). The results demonstrate that all compounds (F1, F2, F3, and F4) exhibited dose-dependent efficacy in reducing glutamate-induced pain, with the first three compounds demonstrating the most pronounced effect. Thus, at the doses of 5, 10, and 20 mg/kg, compound F1 significantly diminished the pain reaction by 41.3% (*p* = 0.0135), 82.3% (*p* < 0.0001), and 87.2% (*p* < 0.0001), respectively; compound F2 significantly diminished the pain reaction by 54.3% (*p* = 0.0002), 62.9% (*p* < 0.0001), and 68.7% (*p* < 0.0001), respectively. Compound F3, at the doses 10 and 20 mg/kg, significantly reduced the pain reaction by 61% (*p* = 0.0344) and 70% (*p* = 0.0163), respectively. The administration of compound F4 at a dose of 20 mg/kg resulted in a significant reduction in pain of 53.7% (*p* = 0.027).

In the capsaicin-induced pain test, the compounds F1, F2, F3, and F4 at doses of 5, 10, and 20 mg/kg after *i.p.* administration were tested (Figure 3B). Compounds F1 and F2 statistically significantly and dose-dependently reduced capsaicin-induced neurogenic pain. F1 diminished neurogenic pain by 31.7% (*p* = 0.0343), 40.8% (*p* = 0.0050), and 69.0% (*p* < 0.0001), respectively, and compound F2 reduced the pain by 31% (*p* = 0.048), 56% (*p* = 0.0001), and 74.7% (*p* < 0.0001), respectively. F3 reduced the pain by 48.46% (*p* = 0.0237) only at the dose 20 mg/kg, while F4 reduced it by 42% (*p* = 0.0458) at the dose of 5 mg/kg, by 33.6% (*p* = 0.045) at the dose of 10 mg/kg, and by 57.25% at the dose of 20 mg/kg (*p* = 0.0056).

#### 2.2.3. Carrageenan-Induced Inflammatory Edema and Hyperalgesia

Intraplanar injection of carrageenan significantly induced edema (Table 2). In the control group, the mean paw volume at time 0 was 0.83 ± 0.01 cm^3^. At 1, 2, and 3 h, the volumes were 1.51 ± 0.04 cm^3^, 1.6 ± 0.05 cm^3^, and 2.01 ± 0.05 cm^3^, respectively. Compared to baseline, these were increases of 82%, 117%, and 142%, respectively. In the carrageenan test, the anti-inflammatory (anti-edema) activity of compounds F1, F2, F3, and F4 was examined at doses of 10 and 20 mg/kg, as well as ketoprofen at a dose of 20 mg/kg, at 1, 2, and 3 h after carrageenan administration (Table 2). F1 at a dosage of 10 mg/kg demonstrated a significant reduction in edema volume at 1, 2, and 3 h into the experiment, with decreases of 27.8%, 17.5%, and 17%, respectively. In contrast, the higher dosage of 20 mg/kg resulted in more pronounced reductions in paw edema, with decreases of 38.5%, 37.2%, and 22.1% observed at the same time intervals. Compound F2 at a dose of 10 mg/kg showed a limited anti-edema effect, reducing edema by approximately 9% compared to the control group after 2 h of testing. In contrast, the 20 mg/kg dose effectively reduced edema at 1, 2, and 3 h after carrageenan administration, achieving reductions of 26.8%, 17.7%, and 10%, respectively. Compound F3 at a dose of 10 mg/kg was only active after 3 h of carrageenan administration, reducing swelling by 9.5%. For the 20 mg/kg dose, edema significantly decreased at 1, 2, and 3 h of testing, reaching values of 16.8%, 19.2%, and 20.1%. Compound F4 demonstrated anti-edema activity at both doses of 10 and 20 mg/kg. At the lower dose, it significantly reduced edema by 8.7%, 17%, and 15.5%, respectively, at 1, 2, and 3 h of testing. At the higher dose of 20 mg/kg, edema decreased by 18%, 21.8%, and 21% in the same time intervals. Ketoprofen, utilized as a secondary reference compound, demonstrated a significant reduction in edema formation, inhibiting it by 39%, 47%, and 35.8% over three consecutive hours of the experiment, respectively.

Furthermore, the antihyperalgesic activity of compounds F1, F2, F3, and F4 given at doses of 10 and 20 mg/kg and ketoprofen at 20 mg/kg was investigated at 1, 2, and 3 h after carrageenan administration (Table 3). Injection of carrageenan induced substantial mechanical hyperalgesia. This hyperalgesic response was observed in terms of the pain withdrawal threshold for mechanical stimuli (gram).

For the control group, the baseline value of the mechanical pain-inducing stimulus (in grams) ranged from 130.8 ± 2.4 g to 140.8 ± 2.0 g. After 1, 2, and 3 h, the pain withdrawal threshold induced by mechanical stimuli decreased, indicating the development of carrageenan-induced mechanical hyperalgesia. After 1 h, it ranged from 119.2 ± 0.8 g to 132.5 ± 2.5 g, after 2 h from 119.2 ± 1.5 g to 126.7 ± 1.05 g, and after 3 h the values were between 117.5 ± 1.1 g and 125 ± 1.8 g.

Administration of the F1 compound at a dosage of 10 mg/kg resulted in a 16% increase in pain threshold after one hour, a 19.5% increase after two hours, and a 14.2% increase after three hours of assessment. A more significant effect was noted at a dosage of 20 mg/kg, resulting in increases in pain threshold of 20.2%, 24.4%, and 25% at 1, 2, and 3 h, respectively. The pain threshold remained relatively stable after administration of 10 mg/kg of the F2 compound, indicating a lack of analgesic activity. A higher dose of 20 mg/kg significantly altered the pain threshold, increasing it by 10%, 14%, and 11.3%, respectively. F3 at 10 mg/kg showed significant increases in pain thresholds of 7%, 12.5%, and 10% at 1, 2, and 3 h, respectively. At the higher dose of 20 mg/kg, it showed the strongest effect at hour 2, increasing the pain threshold by 19% compared to the control. At 1 and 3 h, it significantly increased the pain threshold by 11.9% and 18.6%, respectively, compared to the control. The F4 compound administered at a dosage of 10 mg/kg resulted in an increase in pain threshold of 10.5%, 13.4%, and 12.9% at 1, 2, and 3 h post-administration, respectively. When the dosage was elevated to 20 mg/kg, a significant enhancement in pain threshold was observed, with increases of 14.4%, 16%, and 14.2% during the corresponding time intervals in comparison to the control group. In contrast, ketoprofen at a dosage of 20 mg/kg exhibited the most pronounced analgesic effect, elevating the pain threshold by 19.8%, 38%, and 33.8% after 1, 2, and 3 h of testing, respectively, relative to the control group.

#### 2.2.4. Streptozotocin-Induced Diabetic Neuropathy 

**Figure 4:**
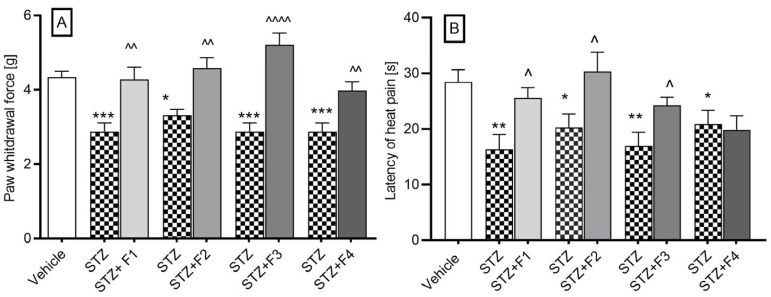
The effect of F1, F2, F3, and F4 compounds administered *i.p.* at the dose of 10 mg/kg, 30 min before the test on tactile allodynia, measured using the von Frey test (**A**), and on thermal hyperalgesia effects assessed in the hot plate test (**B**) in a streptozotocin-induced neuropathic pain model. The results are shown as the mean ± SEM of force applied to elicit paw withdrawal or the mean (±SEM) latency to the pain reaction. Statistical analysis: unpaired Student’s *t*-test. Significance vs. streptozotocin-treated mice: ^ *p* < 0.05, ^^ *p* < 0.01, ^^^^ *p* < 0.0001 and vs. normoglycemic mice^:^ * *p* < 0.05, ** *p* < 0.01, *** *p* < 0.001, n = 10 mice per group. The Shapiro–Wilk test confirmed the normality of the data distribution. STZ- streptozotocin.

The administration of streptozotocin (STZ) led to a reduction in the pain threshold, from a mean value of 4.33 ± 0.17 g (in normoglycemic mice) to a range of values between 2.87 ± 0.23 g and 3.3 ± 0.15 g (in diabetic mice). Furthermore, we evaluated the influence of compounds F1–F4 on diabetic mice with induced allodynia for mechanical stimuli using the von Frey test. All compounds at a dose of 10 mg/kg significantly increased the pain threshold, as illustrated in Figure 4A. Specifically, the pain threshold increased from 2.87 ± 0.23 g to 4.28 ± 0.32 g, reflecting an analgesic activity of 48.6% (*p* = 0.003) following the administration of F1. In the case of F2, the threshold rose from 3.32 ± 0.15 g to 4.58 ± 0.29 g, indicating a 39.4% analgesic activity (*p* = 0.0026). The administration of F3 led to a notable increase from 2.88 ± 0.23 g to 5.21 ± 0.32 g, corresponding to an impressive 81.13% analgesic activity (*p* < 0.0001). Lastly, F4 administration resulted in an increase from 2.88 ± 0.23 g to 3.98 ± 0.23 g, demonstrating a 38.29% analgesic activity (*p* = 0.0039).

Further, in the hot plate test (55 °C), diabetic mice exhibited a significant reduction in pain response latency time, from 28.43 ± 2.21 s to a value ranging from 16.34 ± 2.69 s to 20.89 ± 2.47 s. The 10 mg/kg dose statistically significantly increased the pain response time from 16.34 ± 2.69 s to a value of 25.59 ± 1.84 s after F1 administration, which represented 56.6% analgesic activity; from 20.33 ± 2.38 s to 30.36 s after F2 administration, which represented 49.33% analgesic activity; from 16.96 ± 2.45 s to a value of 24.24 ± 1.46 s, which represented 42.92% analgesic activity after F3 administration. The compound F4 did not affect the latency time of the pain response (Figure 4B).

#### 2.2.5. Oxaliplatin-Induced Neuropathic Pain

We investigated the influence of compounds F1–F4 on oxaliplatin-induced allodynia for mechanical stimuli using the von Frey test. In all experimental groups, administration of oxaliplatin (10 mg/kg) resulted in a statistically significant reduction in the mean paw withdrawal force, assessed at 3 h (early phase of neuropathic pain) and 7 days post-injection (late phase of neuropathic pain).

**Figure 5:**
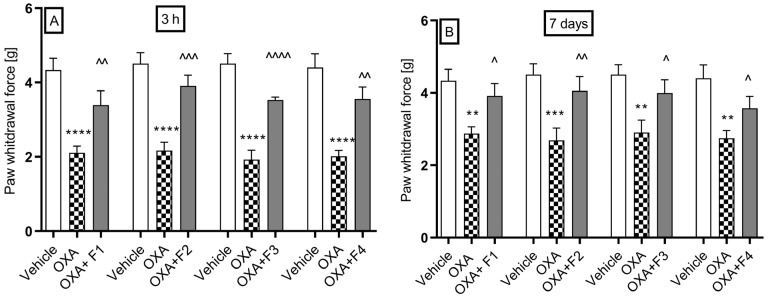
The effect of F1, F2, F3, and F4 compounds on tactile allodynia in oxaliplatin-induced peripheral neuropathy assessed 3 h (**A**) and 7 days (**B**) after oxaliplatin injection measured using the von Frey test. The compounds were administered *i.p.* at the dose of 10 mg/kg, 30 min before the test. Data are presented as mean ± SEM. Statistical analysis: unpaired Student *t*-test: ** *p* < 0.01, *** *p* < 0.001, **** *p* < 0.0001 when results compared to vehicle-treated (1% Tween 80) and ^ *p* < 0.05, ^^ *p* < 0.01, ^^^ *p* <0.001, ^^^^ *p* < 0.0001 when results compared to oxaliplatin-treated group, n = 10 mice per group. The Shapiro–Wilk test confirmed the normality of the data distribution. OXA-oxaliplatin.

The nociceptive reaction in the vehicle group had an average value of 4.44 ± 0.32 g. During the first phase of the test (Figure 5A), the administration of oxaliplatin lowered the pain threshold to values ranging from 1.93 ± 0.25 g to 2.17 ± 0.23 g. A dose of 10 mg/kg caused a change in the pain threshold from 2.11 ± 0.18 g to 3.39 ± 0.46 g, indicating 60.7% analgesic activity after the administration of F1; from 2.16 ± 0.23 g to 3.91 ± 0.29 g (81% analgesic activity) after the administration of F2; from 1.93 ± 0.23 g to 3.53 ± 0.08 g (82.9% analgesic activity) after the administration of F3; and from 2.02 ± 0.16 g to 3.56 ± 0.32 g (76.2% analgesic activity) after the administration of F4.

During the second phase of the test (Figure 5B), the pain threshold significantly decreased to values ranging from 2.78 ± 0.2 g to 2.9 ± 0.34 g. A dose of 10 mg/kg of compound F1 resulted in an increase in the pain threshold from 2.87 ± 0.19 g to 3.9 ± 0.34 g, representing a 35% analgesic activity.

The administration of compound F2 significantly raised the pain threshold from 2.75 ± 0.21 g to 4.0 ± 0.34 g, showing 45.45% analgesic activity. Compound F3 also increased the pain threshold from 2.9 ± 0.34 g to 3.99 g, with 37.59% analgesic activity. Additionally, compound F4 raised the pain threshold from 2.87 ± 0.19 g to 3.57 ± 0.33 g, resulting in 24.39% analgesic activity.

## 3. Discussion

The studies presented in this report are a continuation of our ongoing research on a group of 1H-isoindoline-1,3-dione derivatives, which differ in the type of pharmacophore present in the phenyl ring of the 2-hydroxy-3-(4-aryl-1-piperazinyl)propyl substituent attached to the imide nitrogen atom (compounds R, F1–F3), as well as the 4-benzhydryl analogue F4. These compounds have previously demonstrated potent peripheral antinociceptive effects, a reduction in COX-2 levels, and no cytotoxicity in an in vitro cell line model [6]. Recent studies concerned the estimation of basic pharmacokinetic parameters and the assessment of analgesic and anti-inflammatory activity in different models of pain caused by various thermal and chemical agents that cause similar symptoms of pain, but with different mechanisms of action. The formalin test is a well-established model used to study tonic pain, which refers to persistent, ongoing pain typically associated with inflammatory and pathological conditions, rather than brief, acute pain. Unlike other nociceptive tests (e.g., the hot plate or tail-flick tests), which measure acute phasic pain, the formalin test is unique in that it produces a biphasic pain response [8,9,10]. The first, neurogenic phase corresponds to acute pain and is triggered by the direct activation of primary nociceptive afferents, mainly through TRPA1 channels [11], as well as other mediators [12,13]. The second, late phase represents inflammatory pain, reflecting both peripheral inflammation and central sensitization within the spinal cord [12,14]. Moreover, the neurogenic phase is more responsive to centrally acting analgesics (e.g., opioids), whereas the second phase responds to both centrally and peripherally acting drug] including NSAIDs, corticosteroids, and other anti-inflammatory compounds. In the tonic pain model, the tested compounds showed an analgesic effect, with varying intensity depending on the dose administered and the phase of the formalin test.

In the neurogenic phase, compounds F1 and F2 exhibited the most potent analgesic activity at the highest doses, whereas compounds F3 and F4 were only effective at a dose of 20 mg/kg indicating limited activity in modulating acute neurogenic pain. Compared to ketoprofen, only F1 at the highest dose outperformed ketoprofen’s analgesic effect in the neurogenic phase. These findings are consistent with the pharmacokinetic data, as compound F1, following intraperitoneal administration, exhibited the highest concentration in the brain. Moreover, it demonstrated a favorable brain-to-serum partition coefficient of 1.77, indicative of efficient blood–brain barrier (BBB) penetration. In contrast, the brain concentrations of compounds F3 and F4 were approximately threefold lower. In the inflammatory phase, the most effective compounds, F1, F2, and F3, demonstrated comparable analgesic effects to ketoprofen. The probability of crossing the blood–brain barrier is indicated, among other factors, by the parameter of the topological polar surface area of the tested compounds (TPSA), which ranges from 65.78 to 75.01 A2 (TPSA < 90 A2) [6]. The value of the topological polar surface area of the F4 molecule does not differ from the values calculated for the other imides and is 65.78 Å2, similar to F1 and F3.

In the inflammatory phase, the most effective compounds, F1, F2, and F3, demonstrated comparable analgesic effects to ketoprofen. These results align with earlier findings regarding its anti-inflammatory potential based on COX-2 inhibition and their activity in the writhing test, another model sensitive to inflammatory pain stimuli [6].

Interestingly, compound F4 exhibited a more limited pharmacological profile, showing activity only at the highest dose in both phases of the formalin test and no effect in the hot plate test [6]. These results are more likely attributable to structural differences—such as the presence of the 4-benzhydryl group—that may affect bioavailability, rather than to inhibition of spontaneous locomotor activity (i.e., sedative effects) [6]. These observations are also in agreement with the pharmacokinetic results, as compound F4 exhibited the lowest concentrations in both serum and brain. Given that its brain-to-plasma partition coefficient was comparable to those of the other compounds, the reduced exposure is likely attributable to low bioavailability following intraperitoneal administration. Further pharmacokinetic studies involving intravenous administration of compound F4 may help confirm or refute this hypothesis. Compound F4 did not display sedative properties, in contrast to F1 and F3, which showed mild sedative effects, and F2, which exhibited the strongest sedative activity [6]. The differences in the arylpiperazine pharmacophore structure of compounds F1–F3 are minor: in the case of derivative F2, it is a 2-methoxy derivative, while in position 3 of the aromatic ring of imide F3 there is a strongly electronegative substituent (-CF3). Imide F4 contains a benzhydryl group in the molecule, which can change the surface symmetry of the molecule and its ability to attach to molecular targets. The results of formalin test confirm the analgesic properties of the compounds and suggest that their primary mechanisms of action involve modulation of the inflammatory response and central sensitization, rather than direct inhibition of nociceptor activation. Moreover, their ability to inhibit the late phase of the formalin test—commonly associated with persistent pain states—indicates their potential efficacy in managing chronic pain. These findings are consistent with other preclinical studies on phthalimide-based derivatives, which have demonstrated multi-mechanistic analgesic activity [15].

Although the compounds were active in the phase I formalin test, we decided to evaluate their activity in a capsaicin-induced neurogenic inflammation pain model. Capsaicin acts on sensory neurons (C fibers and Aδ) through direct interaction with the membrane receptor TRPV1 (transient receptor potential vanilloid subtype-1) [16]. Moreover, capsaicin-induced glutamate release activates ionotropic and metabotropic glutamate receptors in the primary afferent fibers [17]. They induce pain-like reactions and rapidly induce a neurogenic pain phase response with burning, local vascular and extravascular reactions, followed by sustained desensitization with prolonged analgesia [18]. Effects caused by the administration of capsaicin resemble the early stage of the formalin stage but depend on TRPV1 rather than TRPA1 activation [8]. In this assay, compounds F1, F2, and F4 (5–20 mg/kg), and F3 (20 mg/kg), reduced capsaicin-induced neurogenic pain, with F3 showing peak efficacy at the highest dose, suggesting possible TRPV1 receptor involvement and the potential for treating TRPV1-mediated pain conditions, including neuropathic and inflammatory pain. Other phthalimide derivatives also exhibited activity in this test [19,20].

From a pharmacokinetic perspective, compound F3 stands out among all tested compounds due to it having the longest half-life in both serum and brain, which correlates with the highest brain AUC value—a key indicator of exposure to the substance. Additionally, compound F3 exhibits the highest brain-to-serum partition coefficient, equal to 2.05. The nociception induced by glutamate is a useful method to evaluate the mechanisms of nociception and the effects of new analgesic drugs [21,22]. Glutamate and its peripheral and central receptors are involved in pain modulation and their activation in specific structures promotes nociceptive responses [23]. In turn, inhibition of glutamate release or receptor blockade results in significant inhibition of acute and chronic pain conditions. Nociception induced by glutamate injection appears to involve peripheral, spinal, and supraspinal sites and may be mediated by ionotropic receptors such as N-methyl-d-aspartic acid (NMDA) and metabotropic glutamate receptors, and also by the TRPV1 receptor and ASICs (acid-sensing ion channels) [21,22,24]. Considering the aforementioned findings and our results, it is conceivable that all compounds may exert their antinociceptive activity, at least in part, through interaction with the glutamatergic system. However, it should be noted that compounds F1, F2, and F3 exhibited the strongest analgesic effects, whereas F4 was noticeably less potent. The limited activity of compound F4 may be attributed to its previously mentioned low bioavailability. Compound F4 exhibited the lowest concentrations in both serum and brain. Given that its half-life was relatively short and comparable to that of compounds F1 and F2, the area under the curve (AUC) calculated for F4 in both compartments was also the lowest among the tested compounds. Also, in this experiment the activity of the benzhydryl derivative F4 differs significantly from the effects induced by the imides F1–F3. 

Since compounds F1, F2, F3, and F4 decreased inflammatory pain in the late phase of the formalin test, we assessed their impact on acute inflammation, including edema and hyperalgesia, using the carrageenan-induced inflammation model in rats. It is a widely used acute inflammation model which mimics the course of pathological processes that develop as a result of mechanical tissue damage (fracture, dislocation, other joint injury), known as sterile inflammation [25]. Unlike formalin and capsaicin, carrageenan does not directly stimulate receptors; rather, it causes the secondary release of inflammatory mediators, such as prostanoids and cytokines, from immunocompetent cells [26]. This promotes local inflammation and hypernociception [27]. Compounds F1–F4 reduced inflammatory edema in the carrageenan-induced model and, as shown previously, also demonstrated efficacy in the acetic acid-induced inflammatory pain model and reduced in vitro COX-2 level [6]. However, compound F1 was particularly effective in reducing edema in the early stages and alleviating inflammatory pain (hiperalgesia) at hour 3, especially at doses of 10–20 mg/kg, producing effects comparable to those of ketoprofen. Compounds F2, F3, and F4 were less effective, with both anti-edematous and analgesic effects observed only at the highest dose. Similar to our findings, other phthalimide-based compounds have shown efficacy in the carrageenan-induced inflammation model, further supporting the anti-inflammatory potential of this chemical class [28].

Neuropathic pain is also one of the most common complications associated with the diabetes [29,30,31] and third-generation platinum-based chemotherapeutic drugs. Furthermore, drugs that are effective in managing neuropathic pain tend to preferentially reduce the amplitude of the second phase observed in the formalin test [32,33]. Neuropathic pain is characterized by abnormal hypersensitivity to stimuli (hyperalgesia) and nociceptive responses to non-noxious stimuli (allodynia) [34,35]. The role of phthalimide derivatives, such as thalidomide and lenalidomide, in the treatment of pain neuropathy is still under investigation and not clearly defined. There is some contradictory information regarding the effect of the phthalimide drugs thalidomide and lenalidomide in the treatment of neuropathies of different etiologies. As demonstrated in several studies, thalidomide has been shown to prevent the development of mechanical allodynia and thermal hyperalgesia in models of STZ-induced neuropathy [36,37]. In addition, thalidomide has also been shown to be effective in models of neuropathic pain induced by spinal nerve ligation [38] and also to relieve inflammatory pain symptoms in neuropathy [39]. However, in other cases, it has been reported that thalidomide [40,41] and its derivatives lenalidomide and pomalidomide cause painful neuropathy [42,43]. Moreover, a single administration of thalidomide and its derivatives, lenalidomide and pomalidomide, evoked mechanical and cold hypersensitivity in mice [44]. Other studies suggest that lenalidomide, a drug that is associated with a lower level of toxicity than thalidomide and which is primarily employed in the treatment of multiple myeloma, might theoretically confer benefits in the management of anti-myelin-associated glycoprotein neuropathy [45,46]. Our results show that all compounds significantly alleviated tactile allodynia, as well as reducing the thermal allodynia (except F4) observed in streptozotocin-induced painful diabetic nephropathy. This finding regarding F4 may be attributable to its apparent inactivity in the hot plate test as demonstrated in previous studies [6], or, alternatively, to its divergent chemical structure.

The mechanism of oxaliplatin-induced neuropathy is associated with neuronal damage, neuronal maladaptive plasticity, and glial activation in brain areas and the spinal cord [47,48,49]. Further, the research findings indicate that sodium, potassium, and calcium ion channels, in conjunction with diverse transient receptor potential families (e.g., TRPA1, TRPM8, and TRPV1), play a pivotal role in the pathophysiology of oxaliplatin-induced neuropathic pain [50]. To our knowledge, our study is the first to report the anti-allodynic properties of a compound F1–F4 containing the phthalimide ring in both the acute and late phases of oxaliplatin-induced neuropathy. In contrast, Batista et al. demonstrated the inhibitory effects of a related compound on symptoms of neuropathic pain in a chronic model involving sciatic nerve constriction [15]. In our chemotherapy-induced neuropathy model, all tested compounds (10 mg/kg) exhibited anti-allodynic activity, with the most pronounced effects observed during the acute phase.

Finally, it should be noted that, similarly to the 3,4-pyridinedicarboximide derivatives, we can state that all three structural elements of the tested compounds affect their biological properties [10,51]: the phthalimide group, the structure and length of the alkyl linker (-2-hydroxypropyl), and the structure and chemical nature of the amine residue. Among the 1H-pyrrolo[3,4-c]pyridine-1,3(2H)-dione derivatives, the strongest analgesic properties in the tests performed were demonstrated by the N-4-(2-hydroxypropyl)piperazinylphenyl derivative, which is an analogue of F1, while analogues F2 and F3, derivatives of 2-methoxyphenyl- and 3-trifluorotolylpiperazine, respectively, were only slightly less active than the leading structure. The introduction of substituents to the aryl ring of the amino residue affects the electron acceptor/donor properties, but they are not always beneficial for the biological effect. The extension of the molecule with another aromatic ring—i.e., the introduction of a diphenylmethyl group to the N-1 position of piperazine—may weaken the ability of compound F4 to penetrate the blood–brain barrier, change the way it is distributed to tissues, and cause the need to administer higher doses of the tested substance in order to obtain an analgesic effect similar to that noted for compounds F1–F3 in the conducted experiments. The benzhydryl group in the imide molecule of F4 may constitute a specific steric obstacle, limiting its ability to attach to molecular targets.

## 4. Materials and Methods

### 4.1. Animals

Adult male Albino Swiss mice (CD-1, 18–25 g, age 3–4 weeks) were used in pharmacokinetic studies (n = 96) and in in vivo pharmacological experiments (n = 476), including the formalin test, glutamate- and capsaicin-induced pain tests, and the neuropathy models. Adult male Wistar rats (Krf: WI (WU); 180–250 g; 3–7 weeks) (n = 84) were used only in the carrageenan-induced edema model. All mice and rats were purchased from the Animal Breeding Farm of the Jagiellonian University Faculty of Pharmacy. All experiments were performed between 9 a.m. and 2 p.m. The experiments were performed by a blinded operator unaware of the drug administration conditions or statistical analyses. All efforts were made to minimize the animals’ discomfort. They were group-housed (maximum 10 animals per cage) under standard conditions (20–24 °C, 45–65% humidity, 12-hr dark/light cycle) in cages provided with environmental enrichment. Animals had free access to food and water. The animal study protocols were approved by the First Local Ethical Committee in Krakow, Poland, nos. 359/2019, 440A/2020, 440B/2020, 441/2020, 730/2023 and experimental procedures complied with the European Union Directive of 22 September 2010 (2010/63/EU) and relevant Polish regulations. In line with the 3Rs (replace, reduce, and refine) guidelines, appropriate measures were taken to reduce the number of animals used in procedures and to minimize animal pain and suffering.

### 4.2. Drugs and Doses

The synthesis of the investigated compounds (F1–F4) was performed in the Department of Medicinal Chemistry, Wroclaw Medical University, and synthesis and preliminary study was described in our previous work [6]; see Figure 1. For the in vivo studies, the compounds were suspended in 1% Tween 80 solution (Merck, Germany) and administered by the intraperitoneal *(i.p*.) route 30 min before the pharmacological experiments, at a constant volume of 0.1 mL/10 g (mice) and 0.1 mL/100 g (rats). The doses of 5, 10, and 20 mg/kg were determined based on data from previous pilot studies with compounds F1–F4 [6], structurally related analogues [25], and relevant findings reported in the literature. Raw experimental data are presented in the figure for clarity, while Section 2 focuses on percentage changes relative to the control group. Control animals were administered an equivalent volume of vehicle (1% Tween 80 solution) via the same route as the test compound. The following reagents and drugs were used: Formalin (37% *w*/*w* formaldehyde solution, P.O. Ch., Gliwice, Poland), capsaicin (Sigma-Aldrich, Darmstadt, Germany), 0.9% saline (Polfa, Kutno, Poland), oxaliplatin (Activate Scientific, Prien am Chiemsee, Germany), streptozotocin (STZ) (Sigma, Kawasaki, Japan), λ-carrageen (Sigma, Kawasaki, Japan), PBS (Perkin Elmer, USA), ketoprofen, and glutamic acid (Sigma, Kawasaki, Japan).

### 4.3. Pharmacokinetic Study

#### 4.3.1. Study Design

The compounds F1, F2, F3, and F4 were suspended in a 1% Tween 80 solution in sterile water for injection and given to mice *i.p.* at a dose of 5 mg/kg (n = 3 per time point; all mice in procedure = 96) as a single injection at a volume of 10 mL/kg. At specific time points (5; 15; 30; 60; 120; 240; 360; and 480 min after compound administration), mice were sacrificed by decapitation under isoflurane (5%) anesthesia to harvest blood and brain for assays. After allowing the blood to coagulate for 20 min at room temperature, blood samples were centrifuged for 10 min at 8000 rpm (Eppendorf miniSpin centrifuge) to obtain serum. Moreover, brains were removed (at the same time point) from skulls and washed with 0.9% NaCl. The obtained serum and brains were stored at −80 °C until analysis.

#### 4.3.2. Sample Preparation and Analytical Method

Concentrations of compounds F1 to F4 in mice brains and serum were measured by the liquid chromatography tandem mass spectrometry (LC-MS/MS) method using a Sciex QTRAP 4500 triple quadrupole mass spectrometer coupled to an Excion LC AC HPLC system (both from Danaher Corporation, Washington, DC, USA). The brains were homogenized in distilled water at the ratio of 1:4 (*w*/*v*) with a tissue homogenizer ULTRA-TURRAX T10 basic (IKA, Königswinter, Germany). The stock solutions of analyzed compounds were prepared in methanol at the concentration of 1 mg/mL. For the preparation of calibration curves, working standard solutions were prepared in methanol by the serial dilution of the stock solution at the following concentrations: 0.005; 0.01; 0.1; 0.25; 0.5; 1; 2.5; 5; and 10 µg/mL. To prepare samples for the calibration curve, 45 μL of matrix (blank plasma or brain homogenate) was spiked with 5 μL of standard working solution at an appropriate concentration level and vortexed for 10 s. Brain homogenates or serum samples (50 µL) as well as calibration samples were deproteinized with 150 μL of 0.1% formic acid in acetonitrile with an addition of internal standard (valsartan), shaken for 10 min (IKA Vibrax VXR, Königswinter, Germany), and centrifuged for 5 min at a speed of 8000× *g* (Eppendorf miniSpin centrifuge, Hamburg, Germany). Then, brain or serum supernatants were transferred into the autosampler vials. The autosampler temperature was maintained at 15 °C and a sample volume of 1 μL was injected into the LC-MS/MS system.

Chromatographic separation was carried out on the Hypersil GoldTM C18 analytical column (2.1 × 50 mm, 3 µm, Thermo Scientific, Waltham, MA, USA) with the oven temperature set at 30 °C. The mobile phase containing 0.1% formic acid in acetonitrile and 0.1% formic acid in water was delivered at a flow rate of 0.4 µL/min in the gradient mode. Electrospray ionization (ESI) in the positive ion mode was used for ion production. The tandem mass spectrometer was operated at unit resolution in the selected reaction monitoring mode (SRM), monitoring the transitions of the protonated molecular ions m/z 366 to 201 (CE = 40 eV) and *m*/*z* 366 to 160 (CE = 40 eV) for compound F1, *m*/*z* 396 to 190 (CE = 39 eV) and m/z 396 to 160 (CE = 49 eV) for compound F2, *m*/*z* 434 to 145 (CE = 95 eV) and *m*/*z* 434 to 160 (CE = 47 eV) for compound F3, and *m*/*z* 456 to 167 (CE = 27 eV) and *m*/*z* 456 to 152 (CE = 85 eV) for compound F4. The first pair was used as a quantifier and the second for the identity verification—qualifier. One pair (*m*/*z* 436 to 235; CE = 42 eV) was monitored for valsartan used as an internal standard. The mass spectrometric conditions were optimized by continuous infusion of the standard solutions at the rate of 7 μL/min using a Harvard infusion pump (Harvard Apparatus, Holliston, MA, USA). The ion source temperature was maintained at 450 °C and the ion spray voltage was set at 4500 V. The curtain gas (CUR) was set at 40 and the collision gas (CAD) at Medium. The calibration curves were constructed by plotting the ratio of the peak area of the studied compound to internal standard (IS) versus drug concentration and generated by weighted (1/x·x) linear regression analysis. They were linear over the concentrations from 0.0005 to 1 µg/mL of serum and from 0.0025 to 5 µg/g of brain tissue for all analyzed compounds. The precision and accuracy values were within the acceptance criteria recommended in FDA and EMA guidelines for bioanalytical method validation. No significant matrix effect was observed and there were no stability-related problems during the routine analysis of the samples. Results showing details of the inter- and intraday accuracy and precision as well as representative chromatograms are included in the Supplementary Materials (see Figures S1–S8 and Tables S1 and S2).

### 4.4. In Vivo Pharmacological Studies

#### 4.4.1. Formalin Test

The pain reaction was induced by intraplantar (*i.pl.*) injection of 20 μL of 2.5% formalin solution into the right hind paw of the mouse (n = 136). Immediately after injection, the animals were placed individually into glass beaker sand and were observed for the next 30 min. Time (in seconds) spent on licking or biting the injected hind paw in selected intervals, 0–5 min (neurogenic phase) and 15–30 min (inflammatory phase), was measured in each group and was an indicator of nociceptive behavior. The procedure was based on the method described by [52,53] with some modifications by [25].

#### 4.4.2. Glutamate Test

Prior to the experimental procedure, individual mice were acclimatized in an observation chamber (n = 128). Thirty minutes before the test, the compounds under investigation were administered intraperitoneally (*i.p*.). A volume of 20 μL of a glutamate solution, containing 30 μmol per mouse paw, was subcutaneously injected into the ventral surface of the right hind paw to induce a pain response [54]. Glutamate-induced nociception was assessed 15 min post-injection. The duration of time spent licking the injected paw was recorded using a chronometer and was interpreted as an indicator of nociception.

#### 4.4.3. Capsaicin-Induced Neurogenic Pain

The examined compounds were administered 30 min before the test (n = 112). The pain reaction was induced by injecting 20 μL of a capsaicin solution prepared in saline (1.6 μg capsaicin per mouse paw) into the dorsal surface of the right hind paw [25,55] for further details. The animals were immediately placed individually into glass beakers and the amount of time spent licking the injected paw was recorded for 5 min with a chronometer.

#### 4.4.4. Carrageenan-Induced Inflammatory Pain and Edema

The acute and local inflammation and paw edema was induced by intraplantar (*i.pl.*) injection of 0.1 mL of 1% carrageenan solution in PBS into the rat’s right hind paw, according to the modified method of Winter [56] and Lence [57], as described previously [10]; (n = 84). The paw volume was measured using the dislocation of the water column of the plethysmometer (Type a, 7140; Plethysmometer, Ugo Basile, Gemonio, Italy) before (V0) and at 1, 2, and 3 h (V1, V2, V3) after *i.pl*. carrageenan injection. The compounds were administered at the highest active dose (20 mg/kg), intraperitoneally (*i.p*.), 30 min prior to carrageenan injection; similarly, ketoprofen (reference compound, positive control) was administered at the dose of 20 mg/kg. Vehicle (1% Tween 80) was administered by the same route as the tested compounds. Table 2 shows the raw data, while the results expressed as the percentage of analgesia were calculated using the following formula: % edema inhibition = [(C − V/C] × 100%, where C is the mean paw volume increase measured 1, 2, or 3 h after carrageenan injection in the control group and V is the mean paw volume increase measured 1, 2, or 3 h after the carrageenan injection in the drug-treated group.

Furthermore, we performed the pain pressure threshold test to assess heightened sensitivity to mechanical stimuli. The experiment was conducted based on the method described by Randall and Selitto [58], with certain modifications [25]. An analgesymeter (AnalgesyMeter 37215, Ugo Basile, Gemonio, Italy) was used to gradually apply increasing pressure (measured in grams) to the plantar surface of the right hind paw of the rat until paw withdrawal occurred. At this point, the intensity of the applied force (in grams) was recorded as the withdrawal threshold. The maximum force intensity was set at 250 g. Carrageenan induces a decrease in the nociceptive threshold in response to a mechanical stimulus, a condition referred to as hyperalgesia. For each rat, paw withdrawal thresholds were recorded both before (0 h) and 1, 2, and 3 h after carrageenan administration (i.e., 3.5 h after intraperitoneal pretreatment with the tested compounds). Table 3 shows the raw data, while the results, expressed as the percentage of analgesia, were calculated using the following formula: % hyperalgesia inhibition = [100 × (T/C)] − 100, where C is the mean pressure (g) in the control group and T is the mean pressure (g) in the drug-treated group. The analgesic effect of the tested compounds was assessed in comparison with the control group (1% Tween 80); the injection was performed intraperitoneally within 30 min of carrageenan administration. An analgesic effect occurs when the compound administered produces a pain response with greater pressure than the control group.

#### 4.4.5. Streptozotocin-Induced Diabetic Neuropathy

The induction of diabetes neuropathy in mice [35,59,60,61] was achieved through the administration of streptozotocin (STZ), dissolved in PBS at a dose of 200 mg/kg in a constant volume of 0.1 mL/10 g of mouse body weight, intraperitoneally; (n = 50). Blood glucose levels were measured immediately prior to the administration of streptozotocin and 14 and 21 days following this administration in order to confirm the presence of hyperglycemia. For this purpose, 5 µL of blood was drawn from the tail vein of the mice using a strip glucose meter (AccuChek Active, Roche, Boulogne-Billancourt, France). Following a 21-day observation period, mice exhibiting glucose levels in excess of 300 mg/dL were designated as diabetic and incorporated into subsequent phases of the investigation.

Furthermore, to assess the response to mechanical stimuli (i.e., tactile/mechanical) allodynia), the von Frey test was carried out with application of the electronic von Frey unit (Panlab, Cornellà de Llobregat, Spain) equipped with a fiber, the distal end of which was applied to the right hind paw of the mouse. The device automatically recorded the pressure force (in grams) applied until the paw withdrawal response occurred. In the absence of pain, an increase in the pressure value was observed. The force at which the animal withdrew its paw was considered the pain threshold for the mechanical stimulus. Measurements were taken before streptozotocin administration (in normoglycemic mice) and again 21 days later, both before and after compound administration, in diabetic mice (glucose level > 300 mg/dL) that had developed mechanical allodynia. On the day of the experiment, each mouse was placed in an observation chamber with a wire mesh bottom and was allowed to habituate for 1 h. After the habituation period, in order to obtain baseline values, each mouse was tested 3 times alternately in each hind paw, allowing at least 30 s between each measurement. Then, the mice were pretreated intraperitoneally with compound or vehicle, and 30 min later the animals were tested again and three measurements were taken, the mean of which was calculated to obtain the mean values after drug administration for each mouse. The anti-allodynic effect was defined as an increase in the pressure force value required to elicit a pain response, compared to the control group.

To assess increased sensitivity to thermal stimuli, we used a hot plate testing apparatus (Panlab/Harvard Apparatus, Cornellà de Llobregat, Spain), following the methodology originally described by [62]. In this procedure, after determining the baseline pain response time, 30 min after drug administration, mice were placed on a hot plate maintained at 55 °C and monitored for pain-related behaviors such as licking their hind paws or jumping. To avoid potential damage to paw tissue, a time limit of 45 s was set. Animals that did not respond within 45 s were removed from the apparatus and given a score of 45 s.

#### 4.4.6. Oxaliplatin-Induced Neuropathic Pain

A single intraperitoneal administration of oxaliplatin, dissolved in 5% glucose solution at a dose of 10 mg/kg, resulted in the induction of peripheral neuropathy, which was observed to occur in two distinct phases: the acute-phase tactile allodynia, which manifested within 3 h of administration, and the late-phase allodynia, which manifested after 7 days, (n = 50) [63]. To assess the response to mechanical stimuli (i.e., tactile/mechanical allodynia), the von Frey test was performed using an electronic von Frey device (Bioseb, Vitrolles, France), as described in Section 4.4.5. In this assay, measurements were taken before and 3 h after oxaliplatin administration, as well as 7 days later. The tested compound and the vehicle were administered intraperitoneally to mice exhibiting mechanical allodynia, as confirmed by a statistically significant reduction in pain threshold. A total of 30 min after administration, the assessment was repeated using the same procedure.

### 4.5. Statistical Analysis

The obtained in vivo data were statistically estimated using one-way analysis of variance followed by Dunnett’s test and Šídák’s multiple comparisons post hoc test or t-Student test with Prism 9 (GraphPad Software, San Diego, CA, USA). They were expressed as the means ± standard error of the mean (SEM), n = 8–10 (mice) or n = 6 (rats). Significance is considered at the *p* < 0.05 level. Pharmacokinetic data acquisition and processing were accomplished using the Analyst version 1.7 software.

## 5. Conclusions

In this study, we investigated the antinociceptive effects of four compounds (F1–F4) containing a 1H-isoindole-1,3(2H)-dione core structure, commonly referred to as phthalimide derivatives. A series of in vivo pain models were employed, including tonic (formalin test), neurogenic (capsaicin and glutamate tests), neuropathic (oxaliplatin-induced peripheral neuropathy and streptozotocin-induced diabetic neuropathy), and inflammatory (carrageenan-induced paw edema) models. All tested compounds (F1–F4) significantly attenuated various types of pain, including tonic, neurogenic, inflammatory, and neuropathic nociception. Based on the results of our experiments, the observed strongest analgesic properties of the F1 derivative allow us to classify this molecule as the leading structure among phthalimide derivatives. Following intraperitoneal administration, compound F1 reaches high serum concentrations and the highest brain concentrations among all investigated compounds. However, due to its short half-life, the AUC values in both brain and serum are lower compared to the other tested compounds. The optimal analgesic properties are provided by the presence of a bicyclic imide group connected via the imide nitrogen atom to a three-carbon 2-hydroxypropyl linker, separating the phthalimide group from the basic amino group of N-phenylpiperazine. The introduction of additional substituents to the benzene ring (F2, F3) and the expansion of the aromatic part of the amino residue (F4) are not beneficial for the observed analgesic properties. The results suggest that the analgesic and anti-inflammatory effects observed are at least partially mediated through the inhibition of cyclooxygenase-2 (COX-2) [6] and blockade of the transient receptor potential vanilloid 1 (TRPV1) receptor. Future studies will focus on elucidating the precise mechanisms of action of compounds F1, F2, F3, and F4. In addition, we plan to include female subjects in subsequent experiments to investigate potential sex-related differences in pharmacokinetics and analgesic efficacy. Additional pharmacokinetic studies assessing parameters such as plasma protein binding, metabolic stability (e.g., the potential formation of active metabolites), and the linearity of pharmacokinetic processes (i.e., dose–response proportionality) may also help elucidate the observed differences in pharmacological activity among the investigated compounds.

## Figures and Tables

**Table 1:** Pharmacokinetic parameters determined by non-compartmental analysis calculated from mean serum and brain concentration values (n = 3) of F1, F2, F3, and F4 compounds after a single intraperitoneal administration to mice at the dose of 5 mg/kg. C_max_—the maximum serum/brain concentration; t_max_—time to reach the maximum serum/brain concentration; λ_z_—terminal elimination rate constant; t_1/2(λz_)—half-life in the elimination phase; CL—clearance; AUC_0–8h_—area under the concentration–time curve from time 0 to 8 h after administration; AUC_0-inf_—area under the concentration–time curve extrapolated to infinity; V_z_—volume of distribution at the elimination phase; MRT—mean residence time; K brain/serum—brain/serum partitioning coefficient.

Pharmacokinetic Parameters	F1		F2		F3		F4	
	Serum	Brain	Serum	Brain	Serum	Brain	Serum	Brain
C_max_ [µg/L(kg)]	595.75	970	699.75	465.38	210.25	202.25	130.58	163.5
t_max_ [h]	0.083	0.083	0.083	0.083	0.083	0.25	0.083	0.25
λ_z_ [h^−1^]	0.73	0.76	0.92	0.77	0.55	0.53	0.86	0.89
t_1/2(λz)_ [h]	0.95	0.91	0.75	0.90	1.26	1.31	0.80	0.78
CL/F [L/h/kg]	23.67	-	16.76	-	17.84	-	48.02	-
AUC_0–8h_ [µg∙h/L(kg)]	205.95	368.04	292.78	230.83	258.81	520.05	101.99	164.50
AUC_0-inf_ [µg∙h/L(kg)]	211.22	374.60	298.26	237.16	280.26	573.90	104.12	168.99
V_z_/F [L/kg]	32.56	-	18.22	-	32.44	-	55.75	-
MRT [h]	1.32	1.36	1.14	1.41	2.8	3.16	1.47	1.63
K_brain/serum_	-	1.77	-	0.79	-	2.05	-	1.62

**Table 2:** Effects of the compounds tested: F1, F2, F3, F4, and ketoprofen (20 mg/kg, *i.p*.) on carrageen-induced paw edema. Statistical analysis: one-way ANOVA, followed by Dunnett’s post hoc test. Data are presented as the means ± SEM, n = 6 rats. Significance vs. control: * *p* < 0.05, ** *p* < 0.01, *** *p* < 0.001, **** *p* < 0.0001 at 1, 2, and 3 h after 1% carrageenan administration. ns-not significant.

Compounds [mg/kg]		Change in Edema Volume (mL)		
		1 h	2 h	3 h
Control		1.51 ± 0.04	1.83 ± 0.04	2.01 ± 0.05
F1	10	1.14 ± 0.05 ****	1.51 ± 0.09 **	1.67± 0.09 *
	20	1.09 ± 0.02 ****	1.15 ± 0.07 ****	1.55 ± 0.09 **
Control		1.57 ± 0.05	1.98 ± 0.02	2.12 ± 0.04
F2	10	1.51 ± 0.04 ns	1.82 ± 0.03 **	1.99 ± 0.02 ns
	20	1.15 ± 0.08 ***	1.63 ± 0.04 ****	1.91± 0.03 ns
Control		1.37 ± 0.03	1.82 ± 0.02	2.09 ± 0.05
F3	10	1.37 ± 0.04 ns	1.70 ± 0.04 ns	1.89 ± 0.03 *
	20	1.14 ± 0.05 **	1.47 ± 0.07 ***	1.67 ± 0.06 ****
Control		1.50 ± 0.04	1.88 ± 0.05	2.19 ± 0.05
F4	10	1.37 ± 0.04 *	1.56 ± 0.06 ***	1.85 ± 0.04 ****
	20	1.23 ± 0.01 ***	1.47 ± 0.05 ****	1.73 ± 0.02 ****
Control		1.51 ± 0.04	1.83 ± 0.04	2.01 ± 0.05
Ketoprofen	20	0.92 ± 0.05 ****	0.97 ± 0.02 ****	1.29 ± 0.05 ****

**Table 3:** Effects of the compounds tested: F1, F2, F3, F4, and ketoprofen (20 mg/kg, *i.p*) on the pain threshold (g) at 1, 2, and 3 h after *i.pl.* injection of 1% carrageenan in rats. Statistical analysis: one-way ANOVA, followed by Dunnett’s post hoc test. Data are presented as the means ± SEM. n = 6 rats. Significance vs. control: * *p* < 0.05, ** *p* < 0.01, *** *p* < 0.001, **** *p* < 0.0001 at 1, 2, and 3 h after 1% carrageenan administration. ns-not significant.

Compounds [mg/kg]		Pain Threshold (g)			
		0 h	1 h	2 h	3 h
Control		130.8 ± 2.4	119.2 ± 0.8	119.2 ± 1.5	117.5 ± 1.1
F1	10	128.3 ± 1.1	138.3 ± 2.1 ****	142.5 ± 2.1 ****	134.2 ± 2.1 ****
	20	130.0 ± 2.9	143.3 ± 2.5 ****	148.3 ± 2.8 ****	146.7 ± 1.1 ****
Control		137.5 ± 1.1	131.7 ± 1.7	124.2 ± 2	125 ± 1.8
F2	10	132.5 ± 2.1	135. ± 1.3 ns	127.5 ± 1.5 ns	130 ± 2.6 ns
	20	132.5 ± 2.8	147.5 ± 2.5 ****	141.7± 1.7 ****	139.2 ± 0.8 **
Control		140.8 ± 2.0	132.5 ± 2.5	126.7 ± 1.05	125 ± 1.8
F3	10	134.2 ± 2.4	141.7 ± 1.7 *	142.5 ± 2.1 **	137.5 ± 1.7 **
	20	135.8 ± 1.5	148.3± 3.3 **	150.8 ± 3.9 ****	148.3 ± 3.1 ****
Control		134.2 ± 0.8	126.0 ± 2.0	125.0 ± 1.8	123.3 ± 1.8
F4	10	131.7 ± 1.7	139.2 ± 2.0 ***	141.7 ± 1.7 ****	139.2 ± 1.2 ****
	20	134.2 ± 1.5	144.2 ± 0.8 ****	145 ± 1.5 ****	140.8 ± 0.8 ****
Control		159.2 ± 8.9	130 ± 2.9	135.8 ± 3.8	133.3 ± 5.3
Ketoprofen	20	130 ± 2.9	155.8 ± 5.2 **	187.5 ± 9.0 ***	178.3 ± 11.7 **

## Data Availability

Data are contained within the article or Supplementary Materials.

## Supplementary Materials

The following supporting information can be downloaded at: https://www.mdpi.com/article/10.3390/ijms26136026/s1.
