# Supplementary material for: Comprehensive Evaluation of 1H-Isoindole-1,3(2H)-Dione Derivatives: Pharmacokinetic Studies and Analgesic Potential in Various Pain Models
Source: Int J Mol Sci. 2025 Jun 23;26(13):6026. doi: 10.3390/ijms26136026 (PMC12249711; doi:10.3390/ijms26136026)
Supplement: Supplementary file 1 [file ijms-26-06026-s001.zip › ijms-3662625-supplementary.pdf]

Supplementary materials

Comprehensive evaluation of 1H-isoindole-1,3(2H)-dione derivatives: pharmacokinetic studies and analgesic potential in various pain models

Anna Dziubina [1], Dominika Szkatuła [2], Łukasz Szczukowski [2], Małgorzata Szafarz [3], Anna Rapacz [1]*

[1] Department of Pharmacodynamics, Faculty of Pharmacy, Jagiellonian University Medical College, 9 Medyczna St., 30-688 Krakow, Poland

[2] Department of Medicinal Chemistry, Wrocław Medical University, 211Borowska St., 50-556 Wrocław, Poland

[3] Department of Pharmacokinetics and Physical Pharmacy, Faculty of Pharmacy, Jagiellonian University Medical College, 9 Medyczna St., 30-688 Krakow, Poland

*Corresponding author: **Anna Rapacz**, Department of Pharmacodynamics, Faculty of Pharmacy, Jagiellonian University Medical College, Medyczna 9, 30-688 Krakow, Poland, e-mail address: a.rapacz@uj.edu.pl (A. Rapacz), ORCID ID: 0000-0002-0190-1682, Telephone: +48 (12) 62-05-530

Serum

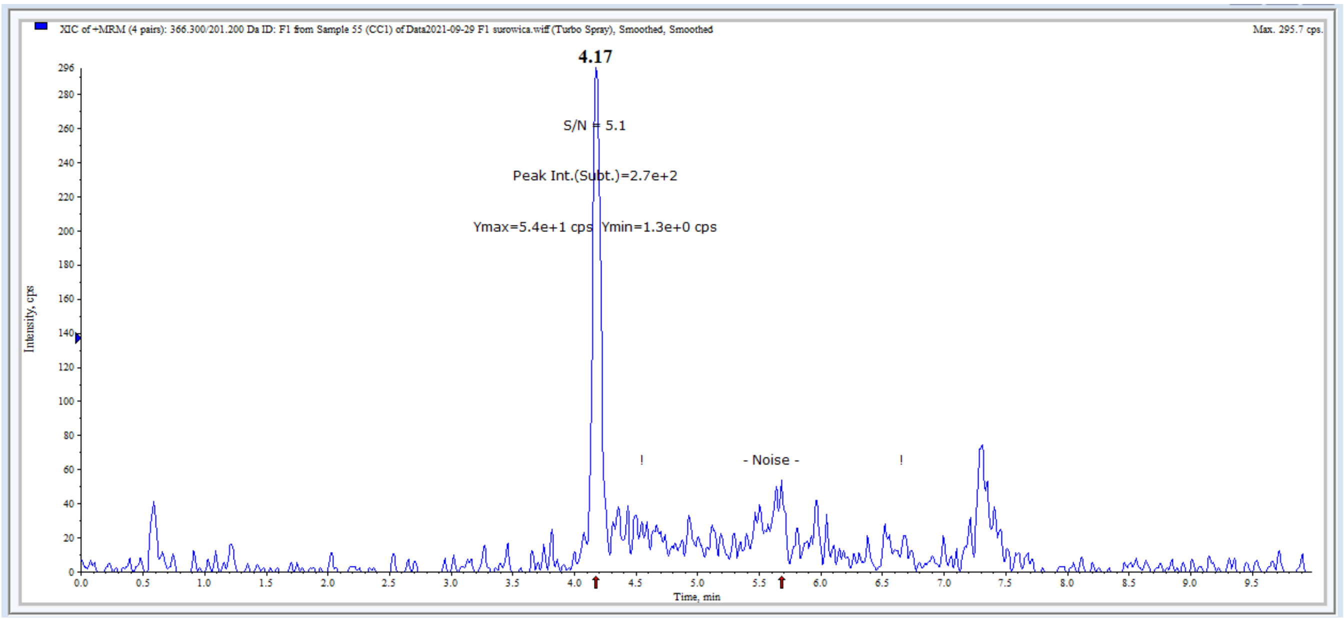

Figure S1. Representative chromatogram of F1 serum calibration sample at the concentration of 1 ng/mL.

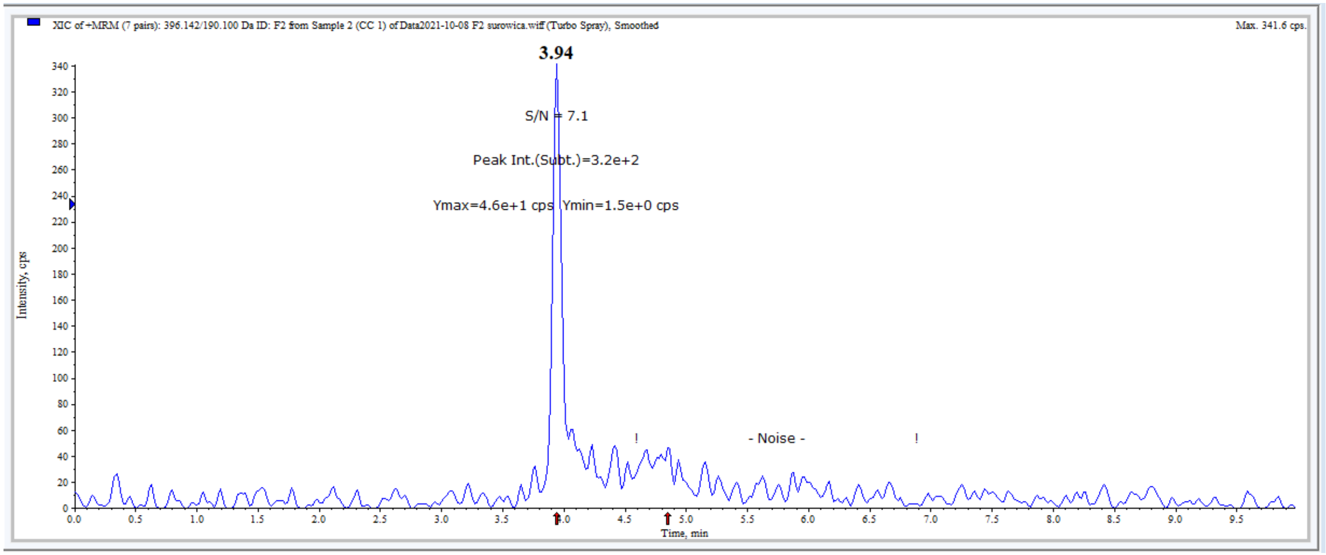

Figure S2. Representative chromatogram of F2 serum calibration sample at the concentration of 1 ng/mL.

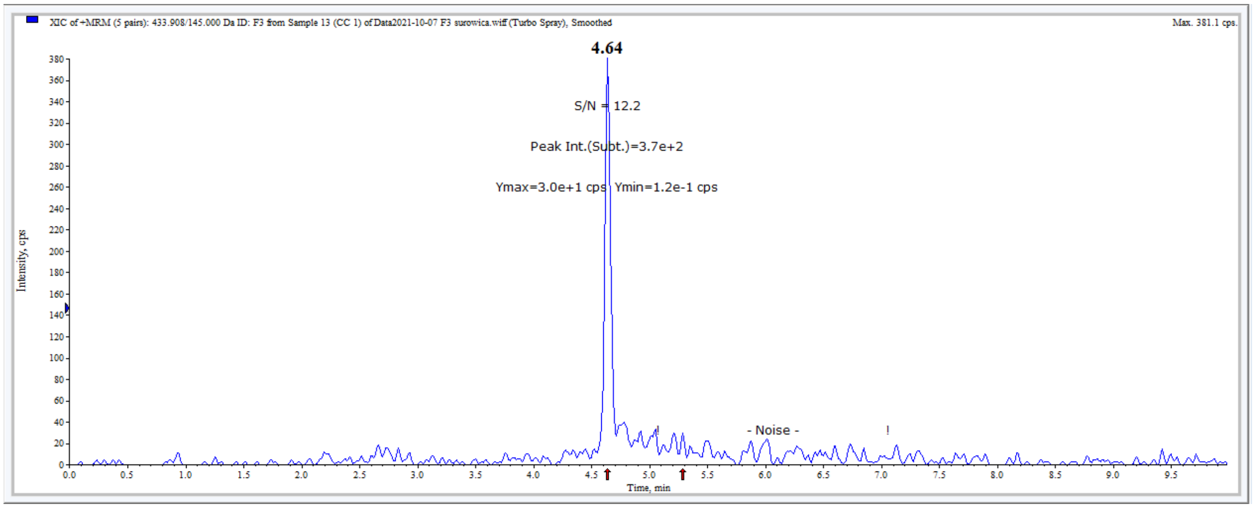

Figure S3. Representative chromatogram of F3 serum calibration sample at the concentration of 1 ng/mL.

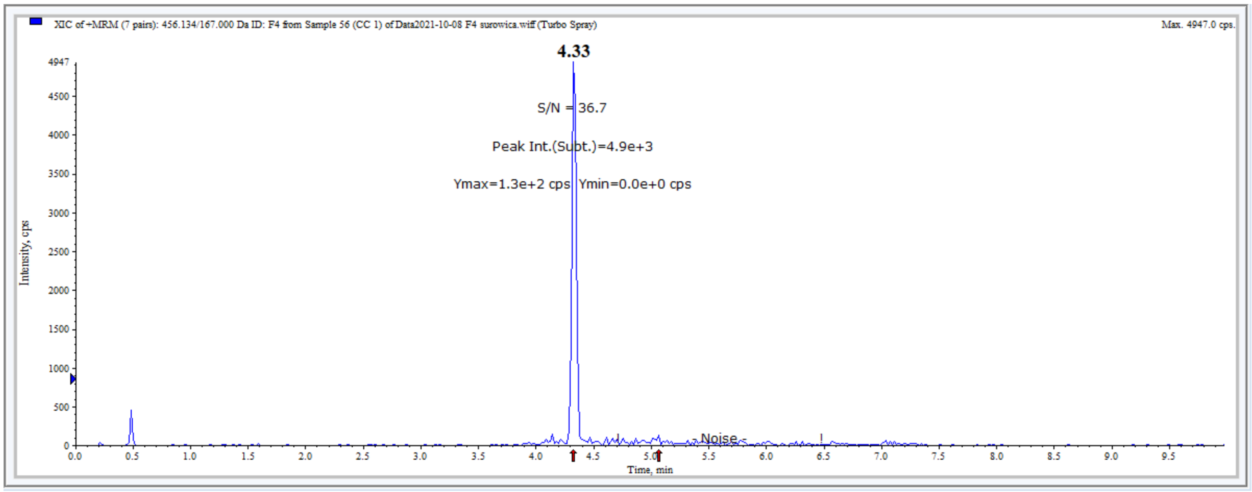

Figure S4. Representative chromatogram of F4 serum calibration sample at the concentration of 1 ng/mL.

Table S1. Intra - and inter - day precision and accuracy of quality control samples (serum).

Analyte	C nominal	Intra - day			Inter - day		
		C measured	CV %	accuracy	C measured	CV %	accuracy
F1	1	0.99 ± 0.027	2.72	99.74	0.99 ± 0.14	14.11	99.49
	25	25.81 ± 1.07	4.15	103.24	25.44 ± 1.66	6.54	108.44
	500	458.63 ± 12.95	2.82	91.73	437.3 ± 12.35	2.82	87.46
	1000	953.5 ± 11.99	1.26	95.35	909.19 ± 11.43	1.26	90.91
F2	1	0.99 ± 0.038	3.82	99.63	1.00 ± 2.92	15.09	100.29
	25	26.63 ± 0.12	0.44	106.51	25.61 ± 1.07	11.39	102.45
	500	528.8 ± 0.5	0.1	105.76	539.39 ± 40.96	7.6	107.88
	1000	987.31 ± 37.2	3.77	98.73	999.89 ± 12.63	1.26	99.99
F3	1	0.99 ± 0.034	3.47	98.81	0.99 ± 0.13	15.07	99.59
	25	23.75 ± 0.33	1.4	95.00	23.72 ± 0.23	0.97	94.89
	500	431.74 ± 0.47	0.11	86.34	475.1 ± 67.73	14.25	95.02
	1000	1010 ± 14.14	1.4	101.00	1004 ± 14.42	1.44	100.4
F4	1	0.99 ± 0.08	8.04	99.72	0.99 ± 0.062	6.23	99.65
	25	25.83 ± 3.52	13.63	103.34	26.35 ± 0.92	3.49	105.5
	500	467.65 ± 0.47	1.64	93.53	480.00 ± 9.83	2.05	95.9
	1000	934.25 ± 19.92	2.13	93.42	967.75 ± 17.97	1.86	96.77

Brain homogenate

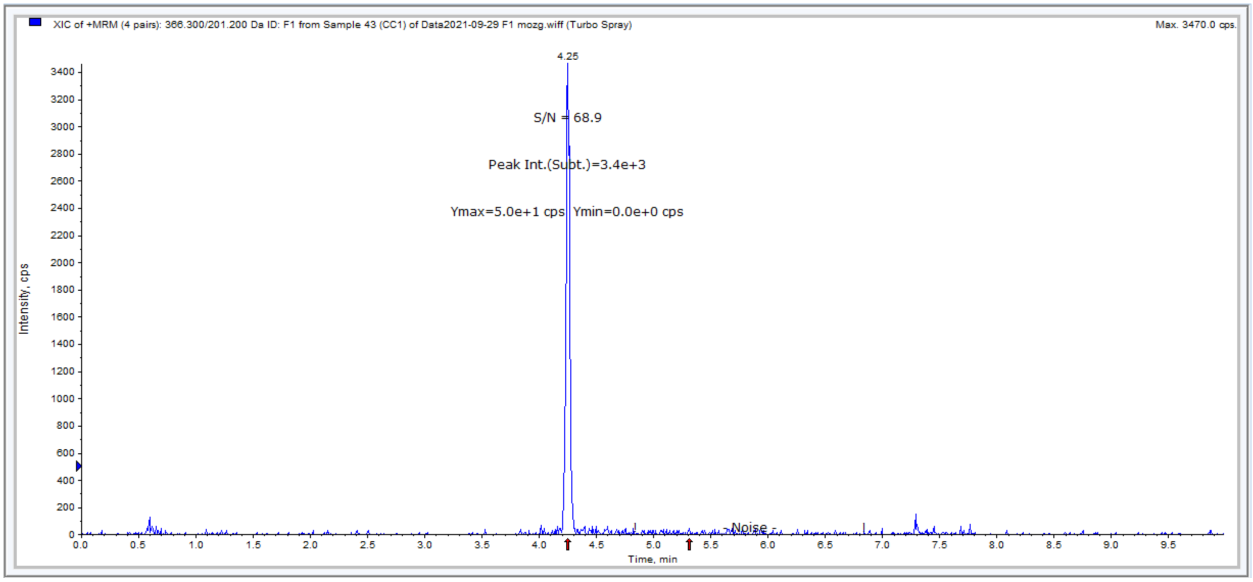

Figure S5. Representative chromatogram of F1 brain homogenate calibration sample at the concentration of 1 ng/mL.

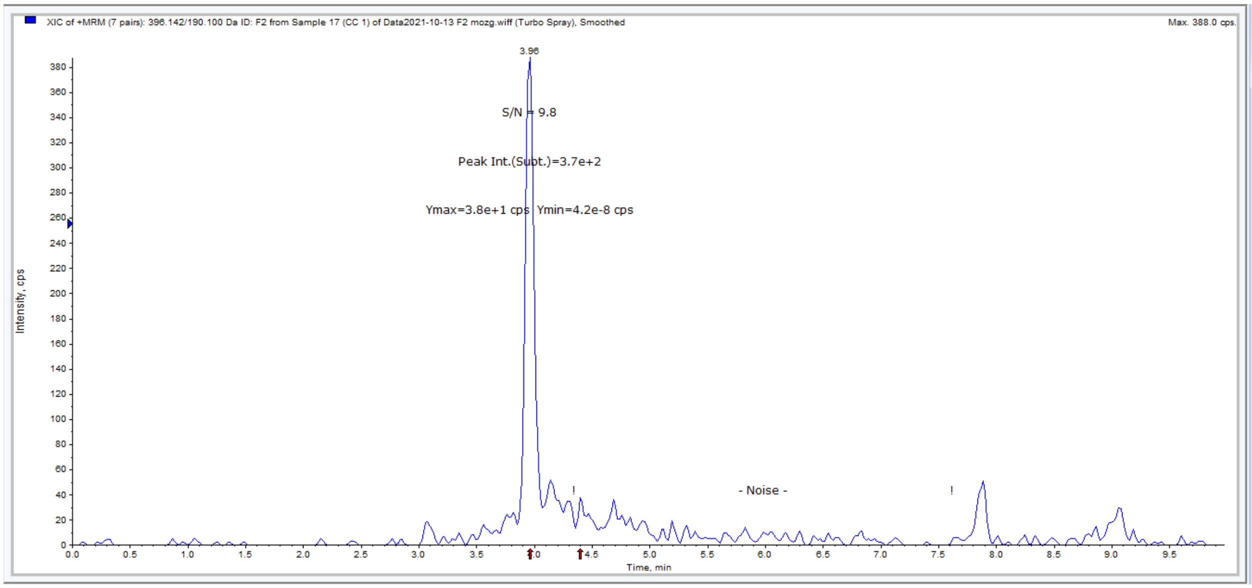

Figure S6. Representative chromatogram of F2 brain homogenate calibration sample at the concentration of 1 ng/mL.

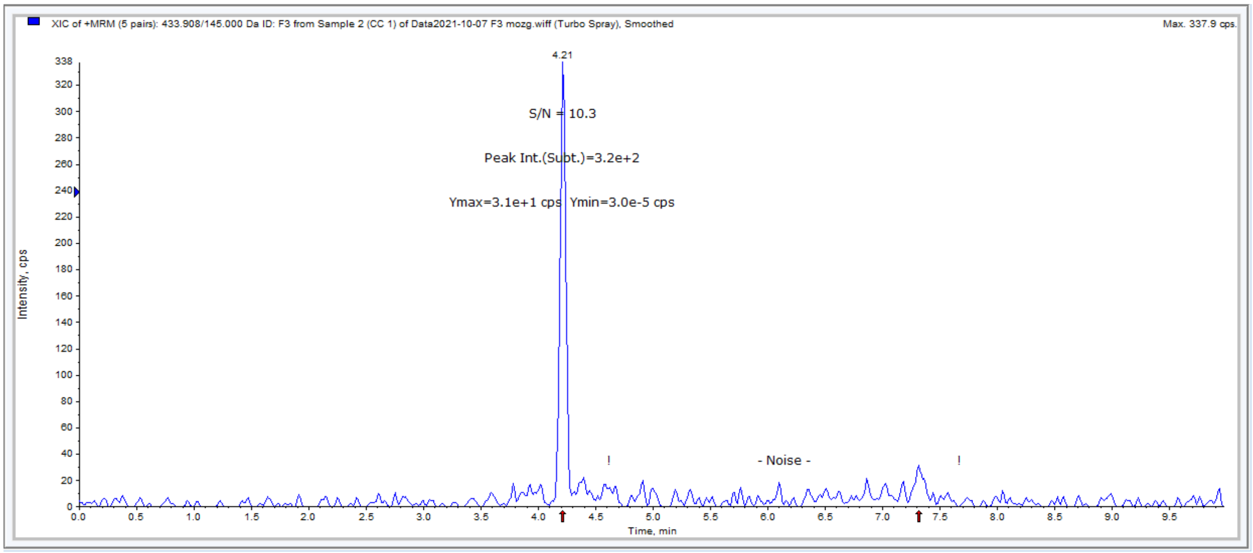

Figure S7. Representative chromatogram of F3 brain homogenate calibration sample at the concentration of 1 ng/mL.

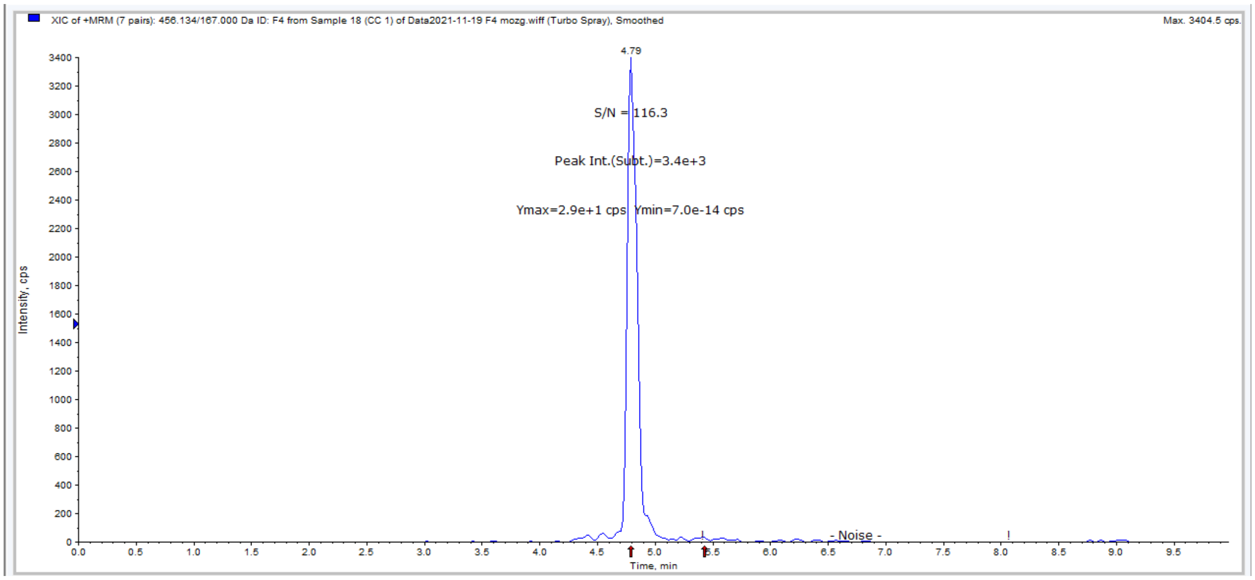

Figure S8. Representative chromatogram of F4 brain homogenate calibration sample at the concentration of 1 ng/mL.

Table S2. Intra - and inter - day precision and accuracy of quality control samples (brain homogenate).

Analyte	C nominal	Intra day			Inter day		
		C measured	CV %	accuracy	C measured	CV %	accuracy
F1	1	0.99 ± 0.025	2.55	99.64	0.99 ± 0.14	15.02	99.28
	25	26.3 ± 2.00	7.6	105.22	25.51 ± 1.89	7.39	102.07
	500	461.31 ± 34.09	7.4	92.26	446.43 ± 33.8	7.57	89.28
	1000	1012.18 ± 150.05	15.01	101.22	966.85 ± 135.18	13.98	97.29
F2	1	0.99 ± 0.015	0.15	99.86	0.99 ± 0.088	8.83	99.85
	25	25.53 ± 1.26	4.93	102.33	26.27 ± 0.92	3.5	105.08
	500	458.33 ± 9.07	1.98	91.67	532.6 ± 3.37	0.63	106.51
	1000	1055.33 ± 82.86	7.85	105.53	993.76 ± 35.97	3.62	99.37
F3	1	0.99 ± 0.047	4.77	99.08	0.99 ± 0.12	12.14	99.42
	25	23.71 ± 0.32	1.34	94.82	23.33 ± 0.57	2.45	93.32
	500	429.35 ± 0.46	0.11	85.87	483.12 ± 69.54	14.4	96.62
	1000	996 ± 5.66	0.57	99.6	1004 ± 14.42	1.44	100.4
F4	1	1.00 ± 0.032	3.23	100.07	0.99 ± 0.019	1.92	99.92
	25	25.83 ± 3.52	13.63	103.34	24.83 ± 3.00	12.12	99.37
	500	516.73 ± 13.72	2.65	103.35	511.75 ± 25.51	4.99	102.12
	1000	855.19 ± 15.08	1.76	85.52	858.25 ± 12.81	1.49	85.82
